# Comparison of speech changes caused by four different orthodontic retainers: a crossover randomized clinical trial

**DOI:** 10.1590/2177-6709.29.3.e2423277.oar

**Published:** 2024-07-08

**Authors:** Diego Coelho LORENZONI, José Fernando Castanha HENRIQUES, Letícia Korb da SILVA, Raquel Rodrigues ROSA, Giédre BERRETIN-FELIX, Karina Maria Salvatore FREITAS, Guilherme JANSON

**Affiliations:** 1 University of São Paulo, Bauru Dental School, Department of Orthodontics (Bauru/SP, Brazil).; 2 Fluminense Federal University, Department of Pediatric Dentistry (Rio de Janeiro/RJ, Brazil).; 3 University of São Paulo, Bauru Dental School, Department of Speech-Language Pathology (Bauru/SP, Brazil).; 4 Ingá University Center, Department of Orthodontics (Maringá/PR, Brazil).

**Keywords:** Orthodontic appliance design, Orthodontics, corrective, Speech, Speech sound disorder, Design de aparelhos ortodônticos, Ortodontia corretiva, Fala, Transtorno fonológico

## Abstract

**Objective:**

This study aimed to compare the influence of four different maxillary removable orthodontic retainers on speech.

**Material and Methods:**

Eligibility criteria for sample selection were: 20-40-year subjects with acceptable occlusion, native speakers of Portuguese. The volunteers (n=21) were divided in four groups randomized with a 1:1:1:1 allocation ratio. The four groups used, in random order, the four types of retainers full-time for 21 days each, with a washout period of 7-days. The removable maxillary retainers were: conventional wraparound, wraparound with an anterior hole, U-shaped wraparound, and thermoplastic retainer. Three volunteers were excluded. The final sample comprised 18 subjects (11 male; 7 female) with mean age of 27.08 years (SD=4.65). The speech evaluation was performed in vocal excerpts recordings made before, immediately after, and 21 days after the installation of each retainer, with auditory-perceptual and acoustic analysis of formant frequencies F1 and F2 of the vowels. Repeated measures ANOVA and Friedman with Tukey tests were used for statistical comparison.

**Results:**

Speech changes increased immediately after conventional wraparound and thermoplastic retainer installation, and reduced after 21 days, but not to normal levels. However, this increase was statistically significant only for the wraparound with anterior hole and the thermoplastic retainer. Formant frequencies of vowels were altered at initial time, and the changes remained in conventional, U-shaped and thermoplastic appliances after three weeks.

**Conclusions:**

The thermoplastic retainer was more harmful to the speech than wraparound appliances. The conventional and U-shaped retainers interfered less in speech. The three-week period was not sufficient for speech adaptation.

## RESUMO

**Objetivo:**

Este estudo teve como objetivo comparar a influência na fala causada por quatro diferentes contenções ortodônticas removíveis superiores.

**Material e Métodos:**

Os critérios de elegibilidade para seleção da amostra foram: indivíduos com 20 a 40 anos de idade, com oclusão aceitável, falantes nativos de português. Os voluntários (n=21) foram divididos em quatro grupos, randomizados com proporção de alocação de 1:1:1:1. Os quatro grupos utilizaram os quatro tipos de contenção, em ordem aleatória e em período integral por 21 dias cada, com período de *wash-out* de sete dias. As contenções superiores removíveis foram: *wraparound* convencional, *wraparound* com orifício anterior, *wraparound* em U e contenção termoplástica. Três voluntários foram excluídos. A amostra final foi composta por 18 indivíduos (11 homens; 7 mulheres) com idade média de 27,08 anos (DP=4,65). A avaliação da fala foi realizada em gravações de trechos vocais, realizadas antes, imediatamente após e 21 dias após a instalação de cada contenção, com análise perceptivo-auditiva e acústica das frequências formantes F1 e F2 das vogais. ANOVA de medidas repetidas e teste de Friedman com Tukey foram utilizados para comparação estatística.

**Resultados:**

As alterações de fala aumentaram imediatamente após a instalação das contenções *wraparound* e termoplástica, e reduziram após 21 dias, mas não para níveis normais. Porém, esse aumento foi estatisticamente significativo apenas para as contenções *wraparound* com orifício anterior e termoplástica. As frequências dos formantes das vogais foram alteradas no momento inicial e, após três semanas, as alterações se mantiveram com as contenções convencional, em forma de U e termoplástica.

**Conclusões:**

A contenção termoplástica foi mais prejudicial à fala do que os aparelhos *wraparound*. Os*wraparounds* convencional e em forma de U interferiram menos na fala. O período de três semanas não foi suficiente para adaptação da fala.

## INTRODUCTION

The retention phase aims to keep the teeth in the correct position after active orthodontic treatment and to counteract relapse, which is the natural tendency for the teeth to return to their initial position. To prevent relapse, some type of orthodontic retainer is usually used. Several forms of retention are cited in the literature, but according to a systematic review^1^, there are no data that scientifically support the clinical choice of retention, that is, there is no important evidence that one type of retention is more efficient in its function than the others.^2^

In the initial retention phase, full-time use of the appliances is usually indicated.^3^ ^,^ ^4^ As most of the maxillary retainers are removable, the patient’s collaboration is required for the success of this phase, and the appliance must be comfortable. Among the aspects that involve comfort, interference in the speech articulation is important, since components of the retainers are located on the palatal surface of the teeth and the palate, and impair the movements of the tongue during speech. Between 10 and 15% of the patients report that speech impairment is a reason for not using the retainers.^5^ The influence of different designs of maxillary retainers in speech has already been the subject of investigation, finding that the reduction of acrylic coverage on the palate minimized damage to pronunciation and increased patient comfort.^6^ In fact, the alteration created in the anterior region of the oral cavity by the Hawley appliance leads to distortions in speech production that can persist for up to three months.^7^ ^-^ ^9^ According to a recent systematic review, there are no prospective randomized clinical studies that assess the influence of the different retention appliances used after orthodontic treatment in speech, with quantitative analyzes.^10^

Therefore, the present study aimed to assess the speech changes caused by different types of maxillary removable orthodontic retainers.

## MATERIAL AND METHODS

### TRIAL DESIGN

This crossover randomized clinical trial involved four groups of volunteers randomized with a 1:1:1:1 allocation ratio. The methodology of this trial followed the CONSORT guidelines for randomized clinical trials. This research was approved by the Research Ethics Committee of the Bauru Dental School (protocol n. 1.198.820). Also, it was registered in the National Clinical Trials Registry (REBEC, identifier RBR-2v3k6r) and the International Clinical Trials Registration Platform (ICTRP, International Clinical Trial Number) of the World Health Organization with the universal trial number (UTN) U1111-1173-6254.

The participants were selected voluntarily from undergraduate and graduate students at the Bauru Dental School, based on the following inclusion criteria: Brazilians, between 20-40 years of age, native speakers of Portuguese; presence of first and second permanent molars; presence of an acceptable occlusal relationship, with a Class I molar relationship (variations up to ¼-cusp Class II or ¼-cusp Class III were accepted).

A speech therapist and an orthodontist performed all clinical exams. Some volunteers were excluded, which had morphological features of the stomatognathic system that could negatively interfere with speech and voice articulation, such as: presence of edge-to-edge bite or anterior or posterior crossbites, anterior open bite, severe overbite (> 50% of the height of the mandibular incisor crown), severe overjet (>4mm), crowding greater than 2mm and generalized anterior diastema; subjects under orthodontic treatment or having completed it less than 12 months ago, minimizing the effect of possible orthodontic relapse on speech production; use any type of maxillary retention in the last 2 months (fixed straight 3x3 bonded retention was accepted); presence of edentulous spaces; craniofacial anomalies; intellectual deficits, syndromes, neurological, psychiatric disorders, smoking, drinking, past laryngeal surgery; presence of alteration of the lingual frenulum, according to Marchesan, Berretin-Felix, Genaro, and Rehder (MBGR) protocol,^11^ based on the measurement of the maximum interincisal distance with the tip of the tongue touching the incisive papilla, divided by the interincisal distance with maximum mouth opening (results less than 0.5 characterized alteration of the frenulum and exclusion of the sample); presence of temporomandibular disorder according to MBGR protocol:^11^evaluation of the presence or absence of TMJ noises and pain on palpation in the TMJ or in the trapezius, sternocleidomastoid, superficial masseter and anterior temporal muscles; presence of pathological vocal alteration, assessed by recording excerpts of speech that included the emission of the vowel “A” sustained for 6 seconds, counting from 1 to 10, and spontaneous conversation of 30 seconds.

Forty-seven subjects were primary selected; 20 were excluded because did not meet inclusion criteria; and 6 decline to participate, remaining 21 subjects. The participants (n=21) were randomly blinded and stratified by sex (numbers were determined for groups and volunteers, blinded by who performed the random allocation), through a computer program in four subgroups with five individuals each. Each subgroup used one of the four types of retainers full-time for 21 days (±1 day), except when eating or sleeping. After this period, they remained without retention for one week (wash-out period) and then used another type of retainer for the same time. This was repeated until the four types of appliances were used. Thus, the crossed design was characterized, where the patients were their controls, to assess the alterations produced in the speech and the perceptions generated by the retainers. 

**Figure 1:**
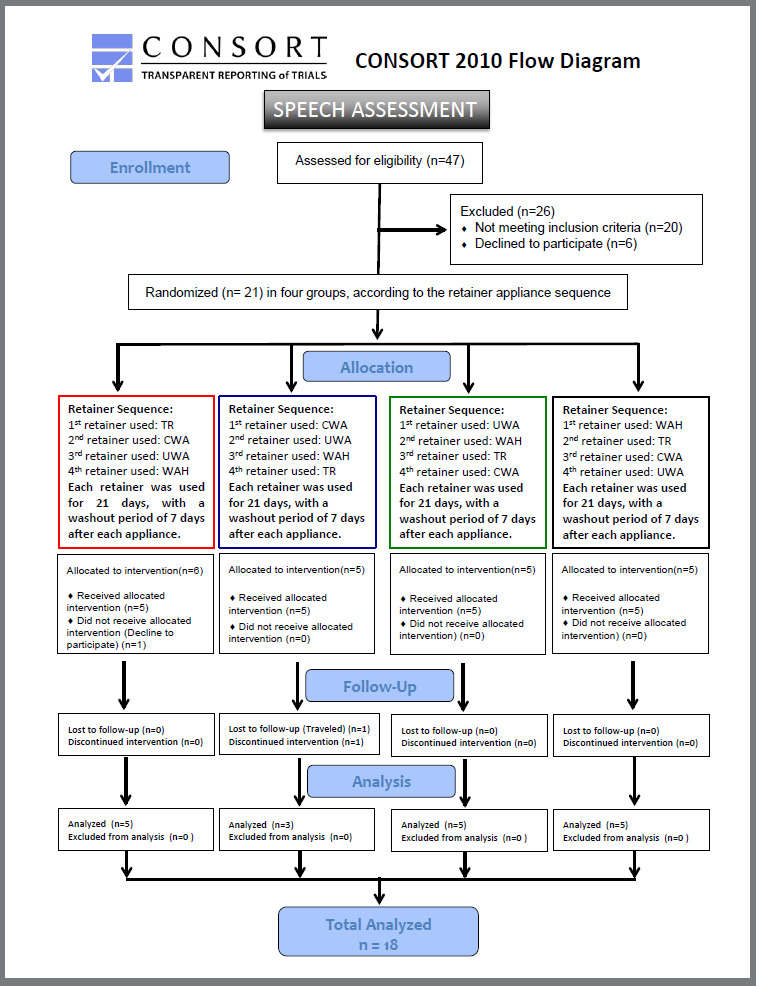
Flow diagram of recruitment and interventions. CWA: Conventional wraparound appliance; WAH: Wraparound with an anterior hole; UWA: U-shaped wraparound; TR: thermoplastic retainer.

Three volunteers did not attend the evaluation appointments and were excluded, as indicated in the flow chart (Fig 1). Eighteen volunteers were included in the final sample (11 male; 7 female) with a mean age of 27.08 years (SD = 4.65). 

## INTERVENTIONS

### RETENTION APPLIANCES USED

All appliances used were made by the same experienced laboratory technician, responsible for the laboratory of the Orthodontics of the Craniofacial Anomalies Rehabilitation Hospital (Centrinho - USP-Bauru, Brazil). The clasps of the wraparound appliances were reused in all appliances of the same patient, changing only the acrylic and minimizing possible interferences. The appliances were made according to the following descriptions.

### CONVENTIONAL WRAPAROUND

**Figure 2:**
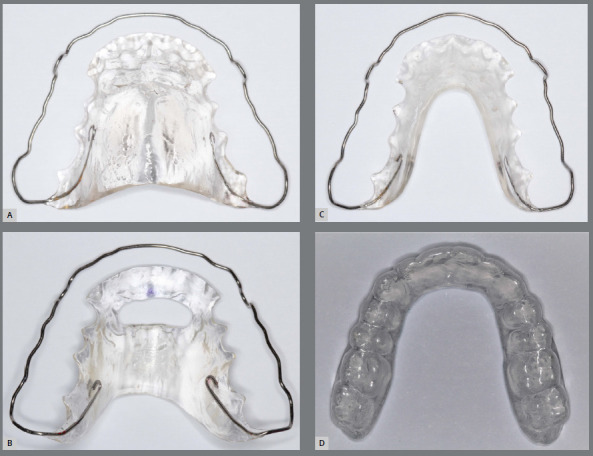
A) Conventional wraparound retainer. B) Wraparound with an anterior hole. C) U-shaped wraparound retainer. D) Thermoplastic retainer.

The wraparound appliance was chosen over the Hawley plate because it does not have clasps around the contact points, generating different occlusal interferences that could impair the evaluation of the effects of the palatal acrylic designs on speech. The conventional design consists of 0.9-mm stainless steel wire clasps and acrylic. The clasp passes through the buccal surface at the middle height of the crowns through all teeth between the two maxillary first molars, and goes around the second molars through the cervical, passing through the distal of these teeth until reaching the palatal surface, where the retention for the acrylic is made. Besides, there is a simple cervical loop in the region between the canine and the first premolar on each side. Acrylic covers the palate with a thickness of approximately 2.5 mm, the anterior limit covers the cervical third of the anterior teeth, the lateral limit is on the cervical of the posterior teeth and the posterior limit is at the height of the distal of the first molar in the region of the palatal raphe to the distolingual of the second molar, for relief of the soft palate (Fig 2A).

### WRAPAROUND WITH AN ANTERIOR HOLE

This type of retainer was included because the hole is located in the region of the palatal wrinkles, an important point of speech articulation and reference for the lingual positioning during swallowing. For this investigation, this model was made with clasps, anterior and posterior lateral limits, and a similar acrylic thickness as the conventional appliance. The anterior hole aims to guide the correct function and lingual posture, as well as to facilitate speech. The approximate width of the acrylic before the hole is 7 mm, and the anteroposterior dimension of the hole is 9-11 mm. The lateral limit of the hole varies according to the projected location of the long axis of the canines, which limits it laterally. The posterior limit is at the mesial of the first molar in the region of the palatal raphe (Fig 2B).

### U-SHAPED WRAPAROUND

Also known as Begg retainer or circumferential retainer. Compared the conventional, it presents alterations in the acrylic, absent in the central portion of the palate. The acrylic is only present in the contour of the teeth, 10 mm width in the posterior region and 12 mm in the anterior, to give more resistance to this region, in addition to presenting slightly greater thickness, but without exceeding 3 mm (Fig 2C).

### THERMOPLASTIC RETAINER

Made with clear plastic material vacuum-formed on the maxillary dental cast, with 1-mm thickness (Essix^®^, Dentsply Sirona, USA). It involves the teeth crowns, from the right to the left maxillary second molar, with a limit of 2 mm above the gingival margin (Fig 2D).

Speech evaluations were performed before (T0), immediately after (T1) and 21 days after the installation (T2) of each appliance. 

### VOCAL RECORDINGS

**Figure 3:**
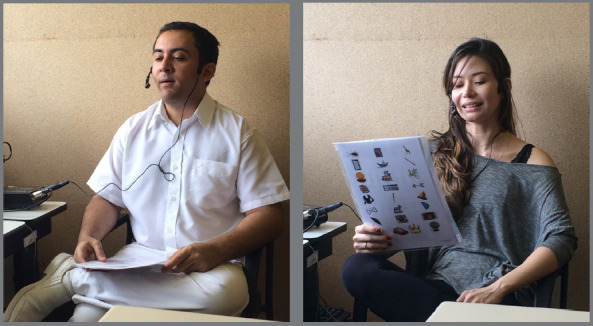
Vocal recordings in an acoustically treated studio.

All participants received prior guidance for each evaluation, to ensure the best production of the exercise. The methodology applied in the collection and analysis of vocal recordings is described below:

» For vocal assessment (auditory-perceptual and acoustic analysis) in the different stages of the research (T0, T1, T2), the voices of all participants were recorded in an acoustically treated studio, directly on a computer using the AKG head microphone, model C444PP, connected to the sound card model Audigy II, Creative brand, positioned 60 degrees from the lip commissure (Fig 3). The recordings were made with the Sound Forge 9.0 program (Sony), at a sampling rate of 44,100Hx, mono channel, in 16 bit.

## SPEECH EVALUATION

### AUDITORY-PERCEPTUAL SPEECH ASSESSMENT

The test followed principles described in the literature, using the MBGR protocol-focused section.^11^ The evaluation included automatic speech (First test - counting from 1 to 10, days of the week, months of the year), naming of phonetically balanced figures, and an excerpt from a spontaneous conversation of approximately 40 seconds. Subsequently, two speech therapists with experience in speech analysis analyzed the recordings, characterizing the presence or absence of distortions in the phonemes. The presence of alterations was recorded with scores that summed, showed results from 0 to 17. Thus, the patient who did not present any alteration received a score of 0, and the one that showed most changes in speech scored 17. The speech therapists were blinded regarding the phase and type of retainer used. The analyzes of each evaluator were compared and, in cases of divergences, they entered into consensus, which was used in the statistical analysis to compare the effect of each retainer on the speech articulation, as well as the possible differences between the retainers.

In this part, the changes analyzed were:

» Omission: when the phoneme is not produced where it should occur, there is no substitution for another sound.
» Substitution: When one sound is replaced by another. In general, substitutions follow the principle of simplification, that is, a more difficult articulation is replaced by an easier one.
» Distortion: When the production of a certain sound is altered so that its result is only approximated to the desired sound. The distortions are, in general, more regular than the two previous types.
» Resonance: It consists of the reinforcement of the intensity of sounds of certain frequencies, being formed by the so-called resonance boxes, which consist of a series of structures and cavities of the vocal apparatus: lungs, larynx, pharynx, mouth, nasal cavity and paranasal sinuses. Ideally, it is balanced, but it can be hypernasal, hyponasal, or laryngopharyngeal.
» Pneumo-phono-articulatory coordination: It is present when there is harmonic coordination between breathing, phonation and articulation; and absent when the individual did not present this harmonic coordination. It is nothing more than the coordination between our breathing and what we say. This coordination is observed during a spontaneous conversation, during reading aloud, or any activity that uses the voice as an instrument. Failure to coordinate this movement can lead to vocal and respiratory fatigue, as well as compromising speech understanding.

## ACOUSTIC ANALYSIS

**Figure 4:**
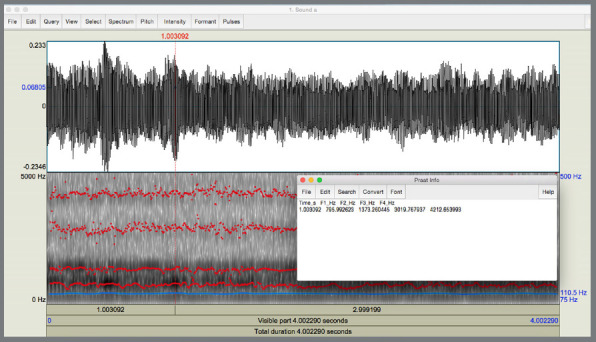
Entries of the software (Praat 5404) used for the analyzes. Edited excerpt of 4 seconds of the vowel “A”, showing the registration of the formants within 1 second.

The acoustic characteristics of the vowels “A”, “E”, “Ê”, “I”, “O”, “Ô”, “U” was evaluated, based on their formants F1 and F2. The individuals were instructed to pronounce each vowel in a medium tone, and the recorded data were saved for later analysis. The excerpts stored in the software PRAAT 5404 (developed by Paul Boersma and David Weenink, at the University of Amsterdam - Netherlands) were stored, edited and evaluated free of charge at www.praat.org (Praat 4.4.33, Institute of Phonetic Sciences, University of Amsterdam, Netherlands) (Fig 4).

Excerpts of approximately 6 seconds of the production of the vowels were recorded, edited to discard the first and last seconds, and only the central 4 seconds were used to obtain the formants. In these 4-second stretches, the formants F1 and F2 were collected in the times of 1s, 2s and 3s, and the average of these formants in these three times were used in the analysis in all phases of the study. The evaluator who performed the editing and collection of the formants was blinded about the stage or appliance. With data obtained, the effect of each retainer on the frequency of the vowel formants was compared, as well as the possible differences between the retainers.

### OUTCOMES

There were no outcome changes after trial beginning. Primary outcomes were the speech changes evaluated by the auditory-perceptual speech assessment and the acoustic analysis.

### SAMPLE SIZE CALCULATION

A sample size calculation was performed based on data from the formant F2 of similar research.^7^ Thus, as an estimate of the standard deviation, an average of the standard deviations for F2 found in this research was considered, which was 280, alpha error of 5%, beta error of 20% and minimum difference to be detected of 270, reaching the value of n = 18 patients in each group. 

### BLINDING

Blinding was performed only for the randomization of the patients for allocation in the four groups. However, patient blinding was not possible.

There was blinding of the evaluators regarding which appliance was being used and the time of evaluation was performed.

### ERROR STUDY

For the error study of the acoustic analysis of vowel formants, 20% of the sample was randomly selected to obtain the formants again. This reassessment was performed with a minimum interval of two weeks between the assessments. Likewise, for the error study of the auditory-perceptual speech assessment, the same 20% of the sample was reassessed by the two evaluators, to perform the intra-examiner error. To assess the inter-examiner error, 100% of the analyzes performed were compared. Dahlberg’s formula was used to calculate the casual error and the dependent *t*-test, for evaluation of the systematic error.

## STATISTICAL ANALYSIS

Shapiro-Wilk test was used to check data normality. The scores of the auditory-perceptual speech assessment and the values of the frequency of the formant F1 and F2 of each retention appliance were compared between the different times evaluated and between the retainers in each time, using the repeated measures ANOVA or Friedman’s test, with Tukey’s *post-hoc* test. The tests were performed using the SigmaPlot 12 program (Systat Software Inc., San Jose, CA, USA). Results with *p*<0.05 were considered statistically significant.

## RESULTS

There was no significant intra or inter-examiner systematic error, and the casual errors were minimal and considered acceptable.

For all appliances, the scores revealed that speech changes increased immediately after installation (T1) of the appliances and decreased after 21 days (T2), but without returning to the levels prior to installation (T0). However, the increase in speech changes at T1 was statistically significant only in the wraparound with anterior hole and in the thermoplastic retainer, and the decrease in these changes in T2 was not sufficient to eliminate the statistical significance in relation to T0 (Table 1). Also, the changes in speech caused by the thermoplastic retainer immediately after installation (T1) were significantly greater than those caused by the other appliances (Table 1).

**Table 1:** Results of the comparison of the auditory-perceptual speech assessment between the different appliances and times evaluated (repeated measures ANOVA and Tukey tests).

Appliances/Times	T0 Before Mean (SD)	T1 Immediately after Mean (SD)	T2 21 days after Mean (SD)	p-value
Conventional WA	2.88 (1.56)	3.72 (0.89) ^A^	3.50 (1.79)	0.121
WA with anterior hole	2.22 (1.47) ^a^	3.83 (1.20) ^Ab^	3.67 (1.32) ^b^	< 0.001*
U-shaped WA	3.00 (1.32)	3.83 (1.33) ^A^	3.44 (1.85)	0.160
Thermoplastic retainer	2.55 (1.72) ^a^	4.88 (1.49) ^Bb^	3.83 (1.58) ^b^	< 0.001*
p-value	0.153	0.005*	0.804	

* Statistically significant for p<0.05. WA = wraparound.

Different lowercase superscript letters in the same row indicate the presence of statistically significant difference between the times of evaluation, indicated by the Tukey test.

Different uppercase superscript letters in the same column indicate the presence of statistically significant difference between the appliances evaluated, indicated by the Tukey test.

In general, the frequencies of the formants F1 and F2 of the vowels were slightly changed in the different appliances and times evaluated (Tables 2, 3 and 4). In the conventional wraparound, the F2 of the vowels “Ó” and “U” slightly increased in T1, and reduced to normal levels in T2, but only with a significant difference for the reduction from T1 to T2 (Table 3). In the U-shaped wraparound, the frequency of the formant F2 in the vowel “Ê” increased significantly in T1 and increased again in T2. In the vowel “I”, F1 increased significantly at T1 and returned to normal levels at T2 (Table 2). In the vowel “U”, F2 decreased slightly in T1 and decreased more in T2, with a significant difference from T0 (Table 3). In the thermoplastic retainer, the F2 of the vowels “A” and “É” decreased in T1 when compared to T0 and remained low in T2, with statistically significant difference only in T2 (Table 3).

**Table 2:** Results of the comparison of the frequency of the formant F1 of the vowels.

VOWEL	FORMANT F1						p-value
	T0		T1		T2		
	Mean (SD)	Median (IR)	Mean (SD)	Median (IR)	Mean (SD)	Median (IR)	
CONVENTIONAL WRAPAROUND							
“A”	765 (138)	722 (162)	777 (115)	762 (184)	756 (120)	693 (185)	0.594^α^
“Ê”	407 (60)	396 (50)	401 (56)	394 (98)	429 (60)	407 (63)	0.064^α^
“É”	580 (81)	570 (49)	576 (63)	570 (94)	569 (60)	561 (76)	0.658^α^
“I”	364 (92)	332 (121)	362 (68)	341 (119)	368 (79)	348 (147)	0.937^α^
“Ô”	463 (51)	442 (89)	483 (78)	457 (96)	465 (61)	456 (59)	0.515^α^
“Ó”	592 (52)	577 (89)	604 (72)	624 (103)	605 (57)	614 (113)	0.717^α^
“U”	412 (68)	406 (118)	414 (74)	431 (129)	456 (65)	444 (112)	0.051^α^
WRAPAROUND WITH ANTERIOR HOLE							
“A”	726 (120)	701 (180)	713 (115)	712 (161)	737 (121)	682 (162)	0.519^α^
“Ê”	416 (59)	410 (54)	417 (37)	417 (68)	415 (39)	419 (63)	0.842^Ϝ^
“É”	565 (70)	546 (106)	553 (92)	554 (108)	576 (46)	583 (50)	0.240^α^
“I”	340 (66)	334 (49)	370 (84)	370 (159)	356 (58)	350 (91)	0.278^Ϝ^
“Ô”	469 (62)	459 (72)	479 (66)	462 (103)	446 (38)	453 (46)	0.411^Ϝ^
“Ó”	594 (56)	585 (70)	599 (61)	588 (101)	592 (49)	582 (72)	0.905^α^
“U”	434 (61)	445 (89)	466 (70)	463 (92)	446 (66)	428 (102)	0.388^α^
U-SHAPED WRAPAROUND							
“A”	727 (112)	684 (183)	721 (132)	722 (172)	736 (86)	736 (151)	0.348^Ϝ^
“Ê”	412 (52)	405 (66)	404 (44)	400 (85)	404 (35)	404 (44)	0.658^α^
“É”	576 (68)	551 (73)	583 (69)	577 (105)	583 (45)	592 (55)	0.861^α^
“I”	350 (68)	333 (53)^a^	403 (111)	371 (128)^b^	363 (58)	345 (71)^a^	0.016*^Ϝ^
“Ô”	466 (81)	454 (87)	476 (44)	483 (57)	476 (48)	470 (89)	0.311^Ϝ^
“Ó”	599 (59)	606 (90)	599 (52)	585 (60)	601 (65)	592 (70)	0.986^α^
“U”	477 (77)	492 (129)	454 (67)	467 (125)	465 (72)	457 (124)	0.570^α^
THERMOPLASTIC RETAINER							
“A”	749 (111)	731 (137)	755 (134)	756 (196)	780 (133)	735 (147)	0.395^α^
“Ê”	416 (67)	393 (87)	429 (66)	413 (95)	438 (73)	418 (113)	0.378^α^
“É”	569 (79)	569 (114)	565 (74)	566 (101)	591 (50)	578 (76)	0.245^α^
“I”	361 (87)	348 (155)	348 (71)	318 (113)	366 (82)	345 (146)	0.554^α^
“Ô”	484 (80)	464 (114)	448 (73)	439 (29)	474 (56)	474 (104)	0.203^α^
“Ó”	600 (59)	600 (83)	593 (83)	600 (143)	599 (63)	592 (74)	0.922^α^
“U”	457 (99)	453 (104)	443 (70)	436 (131)	466 (90)	465 (175)	0.569^α^

* Statistically significant for *p*<0.05. ^α^ Repeated measures ANOVA. ^Ϝ^ Friedman test.

Different lowercase superscript letters in the same row indicate the presence of statistically significant difference between the times of evaluation, indicated by the Tukey test.

**Table 3:** Results of the comparison of the frequency of the formant F2 of the vowels.

VOWEL	FORMANT F2						p-value
	T0		T1		T2		
	Mean (SD)	Median (IR)	Mean (SD)	Median (IR)	Mean (SD)	Median (IR)	
CONVENTIONAL WRAPAROUND							
“A”	1430 (197)	1385 (282)	1390 (222)	1345 (399)	1403 (158)	1381 (265)	0.846^Ϝ^
“Ê”	2116 (213)	2062 (228)	2095 (205)	2080 (297)	2135 (214)	2072 (378)	0.391^α^
“É”	1938 (204)	1895 (348)	1954 (232)	1940 (303)	1913 (275)	1892 (353)	0.486^Ϝ^
“I”	2213 (297)	2180 (373)	2207 (247)	2144 (454)	2211 (255)	2101 (340)	0.989^α^
“Ô”	999 (345)	874 (171)	1172 (462)	951 (1008)	985 (268)	910 (159)	0.486^Ϝ^
“Ó”	1032 (222)	949 (167) ^ab^	1141 (284)	1049 (373)^a^	1013 (122)	993 (137)^b^	0.024*^α^
“U”	1291 (331)	1290 (596)^ab^	1432 (355)	1562 (511)^a^	1219 (362)	1232 (523)^b^	0.037*^α^
WRAPAROUND WITH ANTERIOR HOLE							
“A”	1403 (156)	1395 (235)	1382 (176)	1321 (236)	1382 (162)	1344 (234)	0.663^α^
“Ê”	2094 (195)	2060 (265)	2101 (272)	2050 (470)	2099 (244)	2040 (277)	0.486^Ϝ^
“É”	1944 (257)	1881 (465)	1942 (232)	1853 (458)	1944 (244)	1889 (503)	0.996^α^
“I”	2147 (266)	2133 (203)	2139 (272)	2147 (346)	2176 (213)	2098 (301)	0.679^α^
“Ô”	1093 (318)	952 (583)	1079 (332)	949 (365)	1008 (269)	906 (272)	0.211^Ϝ^
“Ó”	987 (150)	968 (131)	1016 (124)	990 (101)	967 (74)	959 (140)	0.301^Ϝ^
“U”	1278 (396)	1306 (593)	1420 (522)	1327 (878)	1243 (329)	1259 (463)	0.270^α^
U-SHAPED WRAPAROUND							
“A”	1372 (199)	1342 (303)	1361 (144)	1321 (166)	1353 (166)	1308 (297)	0.812^α^
“Ê”	2153 (237)	2116 (436)^a^	2021 (281)	1975 (357)^b^	2100 (209)	2068 (272)^a^	0.006*^Ϝ^
“É”	1939 (250)	1866 (458)	1925 (234)	1873 (264)	1915 (243)	1884 (424)	0.741^α^
“I”	2129 (284)	2121 (225)	2133 (227)	2124 (267)	2156 (246)	2094 (326)	0.883^α^
“Ô”	1114 (365)	941 (636)	1121 (361)	995 (428)	1033 (331)	897 (225)	0.486^Ϝ^
“Ó”	1054 (260)	990 (172)	1022 (162)	965 (158)	1016 (107)	992 (150)	0.678^Ϝ^
“U”	1503 (369)	1459 (369)^a^	1324 (394)	1389 (729)^ab^	1269 (397)	1335 (654)^b^	0.018*^α^
THERMOPLASTIC RETAINER							
“A”	1413 (170)	1402 (313)^a^	1395 (196)	1314 (306)^ab^	1344 (171)	1323 (208)^b^	0.011*^Ϝ^
“Ê”	2102 (253)	2031 (404)	2076 (245)	2022 (306)	2117 (294)	2052 (402)	0.431^α^
“É”	1975 (258)	1891 (478)^a^	1915 (250)	1861 (374)^ab^	1895 (209)	1885 (246)^b^	0.039*^α^
“I”	2166 (249)	2184 (330)	2148 (379)	2186 (375)	2144 (270)	2145 (238)	0.678^Ϝ^
“Ô”	1046 (410)	880 (378)	1023 (443)	901 (154)	971 (262)	879 (146)	0.678^Ϝ^
“Ó”	948 (114)	929 (174)	975 (136)	954 (89)	1033 (208)	968 (182)	0.211^Ϝ^
“U”	1436 (591)	1317 (845)	1446 (449)	1420 (677)	1437 (523)	1263 (840)	0.989^α^

* Statistically significant for p<0.05. ^α^ Repeated measures ANOVA. ^Ϝ^ Friedman test.

Different lowercase superscript letters in the same row indicate the presence of statistically significant difference between the times of evaluation, indicated by the Tukey test.

The frequency of the vowel formant practically did not differ between the appliances at all times evaluated. The only significant difference was found between the thermoplastic retainer in the F1 frequency of vowel “A”, which was significantly higher in T2 than in the wraparound with an anterior hole (Table 4).

**Table 4:** Results of the comparison between the appliances in the times T0, T1 and T2.

VOWELS	T0	T1	T2
	p-value	p-value	p-value
FORMANT 1			
“A”	0.051^Ϝ^	0.931^Ϝ^	0.017*^Ϝ^
“Ê”	0.936^α^	0.267^α^	0.124^α^
“É”	0.827^α^	0.357^Ϝ^	0.255^α^
“I”	0.926^Ϝ^	0.309^Ϝ^	0.863^α^
“Ô”	0.844^Ϝ^	0.119^Ϝ^	0.274^α^
“Ó”	0.957^α^	0.962^α^	0.858^α^
“U”	0.108^α^	0.144^α^	0.843^α^
FORMANT 2			
“A”	0.402^Ϝ^	0.997^Ϝ^	0.153^α^
“Ê”	0.256^α^	0.449^Ϝ^	0.291^Ϝ^
“É”	0.801^Ϝ^	0.610^α^	0.289^Ϝ^
“I”	0.519^Ϝ^	0.526^Ϝ^	0.247^Ϝ^
“Ô”	0.300^Ϝ^	0.413^Ϝ^	0.233^Ϝ^
“Ó”	0.926^Ϝ^	0.161^Ϝ^	0.172^Ϝ^
“U”	0.256^α^	0.501^Ϝ^	0.254^α^

* Statistically significant for *p*<0.05. Tukey test indicated the difference between the thermoplastic retainer and the wraparound with an anterior hole. ^α^ Repeated measures ANOVA. ^Ϝ^ Friedman test.

## HARMS

No serious harm was observed other than the discomfort of using the retainers and the transitory speech changes reported in this study.

## DISCUSSION

This study was a randomized crossover clinical trial, in which only the evaluators were blinded. To date, no known study on the influence of removable maxillary retainers presented this study design. According to a systematic review, there were no prospective randomized clinical studies that evaluated the influence on the speech of different retainers used after orthodontic treatment, through qualitative and quantitative analyzes^10^, exactly what was proposed and performed in this study. Likewise, there were no studies on these influences with a crossover design, where all appliances were used by the same patients, thus being their controls. All studies previously described evaluated the results of retainers in different groups of patients,^6^ ^-^ ^8^ ^,^ ^12^ ^-^ ^18^ a fact that considerably reduces the weight of comparisons, in relation to the crossover design.

The most used removable appliances for maxillary retention were included in this research. Available data confirm that the choice of retainers are the wraparound, the Hawley plate and the thermoplastic retainer, with variations in the order of preference according to the country or region evaluated.^3^ ^,^ ^4^ ^,^ ^19^ ^-^ ^26^ Between the wraparound and the Hawley plate, the choice was the first, because the intention was to assess the influence of acrylic that covers the palate, the component that most directly interferes with speech. Unlike the wraparound, the Hawley plate features Adams clasps that contour the contact points to create retention, and generate occlusal interference that can impair speech and mask the evaluation of the effect of the different acrylic designs proposed.^26^

Since 1967, it has been suggested that more delicate and slimmer maxillary removable retainers devices would generate less speech interference.^9^ ^,^ ^16^ Thicknesses of up to 5 mm have already been described for the wraparound retainer,^6^ but smaller thicknesses were used in this work, balancing appliance resistance and patient comfort, reaching an approximate thickness of 2.5 mm in all wraparound types of retainers, agreeing with previously published data.^18^

Each appliance was used full time for 21 days, except when eating and sleeping. Ideally, the 3-week period is shorter than the standard periods of the retention phase. However, volunteers were not undergoing orthodontic treatment or retention and needed to use the 4 types of retainers, with wash-out periods, which were not feasible in a conventional orthodontic retention phase. As they were volunteers without the need to use retention appliances, long periods of use would be exhausting. Each additional week of use of each of the four retainers would add one month of treatment and, in a prospective clinical study, the dropouts and loss of volunteers for several reasons must be avoided. Even so, there was withdrawal in the initial phase and this research, initially with 21 selected volunteers, was conducted with 18 volunteers.

Also, they did not need to use the retainers during sleep, when speech is not performed and swallowing is reduced. In addition, to avoid bias, the order of use of the retainers was randomized so that all retainer types were used at the beginning, middle and end of the research. The wash-out period of one week was sufficient for the patient to return to the initial patterns before starting the use of the next appliance, since there was no significant difference between the T0 periods (before installation) of the four retainers in the auditory-perceptual and acoustic analyzes.

The time of use was not controlled with daily questionnaires or chips embedded in the appliances,^27^ as it sought to reproduce the clinical routine, which normally does not include these protocols.

The analysis of speech articulation with auditory-perceptual assessment according to the MBGR protocol^11^ was important in the quantification of changes in scores, as it showed differences between the evaluation times of each appliance and also between the types of retainers, as well as the previously proposed methodologies.^7^ ^,^ ^18^ However, the quantification in the MBGR protocol seemed less objective than those already described,^7^ ^,^ ^18^ since these quantified exactly the number of errors in “meaningless” syllables phonetically constructed for the assessment. The MBGR protocol, in general, allows classification and provide changes scores only as absent, systematic or unsystematic, without exactly quantifying the number of errors in the spoken excerpt. On the other hand, it can be applied in stretches of normal speech, including spontaneous, as performed in this study, and incorporates the analysis of resonance, velocity and pneumo-phono-articulatory coordination, a fact that has not been previously demonstrated.

The acoustic analysis of the frequency of the vowel formants proved to be interesting for the study of the changes caused by the appliances in the pronunciation of the vowels, as previously demonstrated.^7^ This analysis is more used especially for vowels, as found in several studies.^28^ ^-^ ^30^ Its application to consonants, whose articulation is more directly influenced by the appliances when compared to vowels, is not so common. A possible explanation is that the application of this methodology to consonants seems more sensitive to errors. In the analysis of the vowels, it is possible to keep the patient emitting the vowel in a sustained way for 6 seconds and, after editing, the central 4 seconds of the stretch in the analysis is used. The duration of the pronunciation of a syllable in a word, that is, of the section to be edited for the evaluation of this syllable, is around 0.2 seconds, sometimes half of this, which makes editing the section and selecting the analysis points more sensitive to errors.

The results of the auditory-perceptual assessment revealed that speech changes increased immediately after the installation (T1) of all retainers, and decreased after 3 weeks of use (T2), but without returning to the initial levels. However, the increase in T1 was only statistically significant for the wraparound with anterior hole and for the thermoplastic retainer, and, even decreasing in T2, remained significantly higher than in T0 (Table 1). These data partially confirm the described premise that retainers with reduced acrylic design on the palate interfere less in speech, compared to those with full acrylic coverage of the palate,^6^ since the results were better in the conventional and U-shaped wraparound appliances than in the wraparound with an anterior hole. An explanation raised for this fact is that more regular areas in the regions of the articulation points of the tongue-alveolar and tongue-palate speech, as in conventional wraparound, would disturb the speech lesser than the irregularity generated in the region of the anterior hole.

Another interesting information was that the speech changes caused by the thermoplastic retainer were statistically higher than those caused by all wraparound retainers. Such data are unprecedented and reflect the series of interferences caused by the occlusal coverage in the occlusion and in the vertical dimension of occlusion, and also in the articulation points of several consonants, especially the labiodental and linguodental ones. Thus, these interferences seem to be more important than those generated by the palatal acrylic of the wraparound at the linguo-alveolar and linguopalatal articulation points of the consonants. This fact also corroborates the findings of the patients’ perceptions, who described the speech interference by the thermoplastic retainer as significantly higher to the other wraparound retainers.^26^ Previous research showed that sound distortion was found with the use of the Hawley and the vacuum-formed retainer.^8^ Still, changes in articulation were more obvious in the Hawley retainer group.^8^ Other recent study demonstrated that the Hawley retainer affected articulatory movements in consonant-vowel combinations more prominently than the thermoplastic retainer.^13^

The reduction of speech changes after three weeks of the use of the appliances confirms the tendency towards normalization and spontaneous gradual adaptation in a relatively short period. However, the fact that the changes after three weeks are still above the initial normal levels reveals that the adaptation is not as fast as previously described, of approximately one week.^18^ These findings are in line with those proposed by the most important known research on speech alteration, which revealed more evident changes in the first evaluated periods, especially after one week of use, and some degree of normalization only after one month and, especially, after three months of use of the retainers.^7^ Even so, the authors observed minor speech disorders in some patients after three months.^7^ However, the design of the present study included a follow-up no longer than three weeks, a period in which speech changes could still be observed.

In general, the frequencies of the formants F1 and F2 of the vowels were slightly changed in the different retainers and times evaluated. This fact was already expected, since there is no articulation of the tongue with teeth, alveolus or palate during the formation of vowels. During this training, the tongue assumes several positions without touching the teeth or palate.^31^

However, there were some changes in the frequencies of the formants F1 and F2 of the vowels, which varied between the retainers. In general, the frequencies of the formant of the vowels increased in T1 and decreased in T2. The change in these frequencies has already been described, specifically in the formants F1 (increase - one week after installation) and F2 (reduction - four weeks after installation).^7^ Thus, it was observed that some vowel formants changed initially with the installation of the retainers and then returned to the initial normal patterns, while others remained altered after three weeks, confirming that this period is insufficient for speech adaptation, as observed in the perceptual assessment and in the literature.^7^

It is known that the frequency of the F1 and F2 formants is directly related to the vertical and anteroposterior position of the tongue, respectively. Thus, F1 increases when the tongue is lower and decreases when higher. F2, on the other hand, increases with the anterior tongue projection and decreases with its more posterior positioning.^32^ Thus, the reduction of F2 in the vowels “A” and “É” with the thermoplastic retainer was possibly due to a more posterior tongue positioning due to the volume occupied by this appliance on the palatal surface of the maxillary teeth. In the same way, the reduction of F2 in the vowels “Ê” and “U” is explained with the U-shaped wraparound. The increase in F1 in the vowel “I” can be explained by the lower position of the tongue with this appliance, since the vowel “I” is an anterior and high vowel, and this plate, in general, had an average thickness slightly greater than the others, so that it presented more resistance in its central portion, but without exceeding the thickness of 3 mm. In the conventional wraparound, on the other hand, the F2 of the vowels “Ó” and “U” initially increased, reducing significantly to T2. The possible explanation for this increase in T1 is that these vowels are high and posterior vowels, especially the “U”, and this retainer has a more posterior acrylic cover than the others, in the region most related to the articulation of the posterior vowels, which led to a more anterior tongue positioning during the articulation of these vowels.

The clinical applications based on the findings of the present study include that the three-week period is not long enough to readapt the speech to the retainers, especially the wraparound with an anterior hole and the thermoplastic retainer. Considering the speech changes, the wraparound retainers interfere less and should be the first choice when the option of maxillary retention is a removable appliance. Although it was not statistically greater than the other wraparound retainers, in general, the U-shaped wraparound showed to be better in the auditory-perceptual analysis and was also the preferred by the patients,^26^ thus being the choice for the orthodontic retention phase.

## LIMITATIONS

Although blinding of the subjects was not feasible, this probably did not impacted the outcomes, since all patients used the four retainers studied, in different sequence. The blinding of the evaluators was performed, since this could influence the results. The three-week period of evaluation is short to allow assessment if the speech changes observed are permanent or transitory. The difficulty in establishing a clinically significant minimum value for the formant in the sample calculation can be a point of discussion. However, the crossover study opposes this by minimizing differences between the groups, as the patient is his own control, which gives rise to the observed differences.

Two patients were excluded from the study because they were not able to attend the appointments at the time needed, but we believe that there is no significant bias due to this missing data, since these losses were not related to the noncompliance in using the retainers, but rather to the missing in the scheduled appointments.

## GENERALIZABILITY

The generalizability of these results might be limited because this research was undertaken in a single center in undergraduate students, and sample size was limited. The study on volunteer patients who were not undergoing orthodontic treatment made it possible to clearly evaluate the variation between the different retainers. However, the crossover study minimizes the differences between the groups, as the patient is his own control, which gives rise to the observed differences. Besides, differences may be observed in patients who are finishing treatment and installing the retainers as they will have immediate previous experience with the devices, potentially reducing or even accentuating the variables observed here. Likewise, the generalization of the data observed here in adults may vary in children and adolescents due to possible differences in adaptation inherent to age.

## CONCLUSIONS

The auditory-perceptual analysis revealed that significant speech changes occurred immediately after the installation of the wraparound with anterior hole and the thermoplastic retainer, and remained after three weeks. The initial change caused by the thermoplastic retainer was significantly greater than the other appliances.

The frequency of vowel formants was impaired by the removable maxillary retainers after installation, and the changes remained present in some appliances after three weeks. The wraparound with an anterior hole was the only one that did not show significant changes in the frequency of the vowel formants.
